# Clinical Value of an LDH–CA125–NLR Panel for Diagnosis, Platinum Resistance, and Prognostic Assessment in Epithelial Ovarian Cancer

**DOI:** 10.3390/curroncol33090551

**Published:** 2026-09-10

**Authors:** Shuiqing Xu, Jinzhen Guo, Ziyi Zhao, Yan Zhai, Yingying Liu, Hua Li

**Affiliations:** 1 Department of Obstetrics and Gynecology, Beijing Chaoyang Hospital, Capital Medical University, 8 Gongti South Road, Chaoyang District, Beijing 100020, China; 2 Chinese Institutes for Medical Research, Capital Medical University, Beijing 100069, China

**Keywords:** epithelial ovarian cancer, lactate dehydrogenase, carbohydrate antigen 125, neutrophil-to-lymphocyte ratio, diagnosis, platinum resistance, prognosis

## Abstract

(1) Background/Objective: Routine blood tests may provide useful information at different stages of epithelial ovarian cancer (EOC) management. This study evaluated continuous lactate dehydrogenase (LDH), carbohydrate antigen 125 (CA125), and neutrophil-to-lymphocyte ratio (NLR) values for diagnostic discrimination in epithelial ovarian cancer (EOC) and, separately, triple-positive status in relation to platinum resistance and survival. (2) Methods: This retrospective study included 238 patients with EOC and 238 individually matched patients with benign ovarian lesions. Continuous biomarkers were entered into logistic regression models for diagnosis. Triple-positive status was defined as LDH > 195.5 U/L, CA125 > 35.37 U/mL, and NLR > 2.63. Logistic regression was used for platinum resistance and Cox regression for progression-free survival (PFS) and overall survival (OS). Bootstrap internal validation was performed for the diagnostic models and PFS nomogram. (3) Results: The continuous LDH + CA125 + NLR model yielded an AUC of 0.873, with 75.97% sensitivity and 88.66% specificity; AUCs were 0.751 in early-stage and 0.958 in advanced-stage EOC. Adding NLR to LDH + CA125 did not significantly improve the AUC overall or within stage groups. Triple-positive status was associated with platinum resistance (adjusted OR = 3.488, 95% CI 1.504–8.087; *p* = 0.004), shorter PFS (adjusted HR = 3.109, 95% CI 1.772–5.456; *p* < 0.001), and shorter OS in advanced-stage EOC (adjusted HR = 3.538, 95% CI 1.385–9.038; *p* = 0.008). The PFS nomogram had a bootstrap-corrected C-index of 0.7521. (4) Conclusions: The continuous three-marker model showed diagnostic discrimination in this selected surgical cohort, while triple-positive status was associated with platinum resistance and adverse survival outcomes. External validation is needed.

## Simple Summary

Ovarian cancer is often found at an advanced stage, and some patients respond poorly to platinum-based chemotherapy. Common blood tests may provide useful information, but each marker alone has limited value. In this study, we evaluated whether continuous values of lactate dehydrogenase, cancer antigen 125, and the neutrophil-to-lymphocyte ratio could be combined in logistic regression models to distinguish epithelial ovarian cancer from benign ovarian lesions. Separately, patients were classified as triple-positive when all three markers exceeded study-specific cut-offs derived from the overall matched cohort, and this status was examined in relation to platinum resistance and survival. The continuous three-marker model showed diagnostic discrimination, particularly in advanced-stage disease, although adding NLR to LDH and CA125 did not significantly improve the AUC. Triple-positive status was associated with platinum resistance and shorter survival. These findings support further evaluation of routine blood-based markers for diagnosis and risk assessment.

## 1. Introduction

Ovarian cancer remains a major cause of morbidity and mortality among gynecological malignancies. According to GLOBOCAN 2022, approximately 324,000 new cases and 207,000 deaths occurred worldwide, while epithelial tumors account for most ovarian cancers and include several histological types with different biological features and clinical outcomes [1,2]. Early epithelial ovarian cancer (EOC) usually causes no specific symptoms, and many patients are diagnosed only after the disease has spread beyond the ovary. However, the difficulty of early diagnosis does not mean that effective population screening is currently available. Long-term follow-up of the UK Collaborative Trial of Ovarian Cancer Screening showed that screening based on serial CA125 measurements or transvaginal ultrasonography did not reduce ovarian and tubal cancer mortality [3]. CA125 is still widely used in clinical evaluation and follow-up, but its sensitivity is limited in early-stage disease, and elevated levels may also occur in benign gynecological and non-gynecological conditions [4].

For patients with newly diagnosed EOC, treatment usually includes cytoreductive surgery and platinum–taxane chemotherapy, followed by maintenance treatment in selected patients according to clinicopathological and molecular characteristics [5,6]. Although most patients initially respond to platinum-based chemotherapy, recurrence remains common, especially in advanced disease; treatment becomes more difficult as sensitivity to platinum decreases. Therefore, simple and readily available indicators that can assist in diagnosis, help identify patients with a higher likelihood of platinum resistance, and provide prognostic information are still needed.

Lactate dehydrogenase (LDH) catalyzes the reversible conversion between pyruvate and lactate and is involved in the metabolic adaptation of cancer cells [7,8]. Increased lactate production can alter the tumor microenvironment, affect immune-cell activity, and promote tumor growth, particularly under hypoxic or nutrient-limited conditions [8,9]. Circulating total LDH is not tumor-specific and can also increase with tissue injury, liver disease, hemolysis, or other systemic conditions; nevertheless, elevated serum LDH has been associated with greater tumor burden and unfavorable outcomes in several cancers [7]. In ovarian cancer, several retrospective studies have suggested that pretreatment LDH may be associated with advanced disease, shorter platinum-free intervals, and poorer survival, although the reported cut-offs and findings have not been consistent [10].

CA125 reflects another aspect of EOC and is routinely used for clinical evaluation, treatment monitoring, and follow-up, although it is not sufficient as a stand-alone diagnostic marker [4]. The neutrophil-to-lymphocyte ratio (NLR), calculated from routine blood counts, provides a simple measure of neutrophil-predominant inflammation in relation to lymphocyte-mediated immune activity. Neutrophils may support tumor growth, angiogenesis, invasion, and immune suppression, but their roles differ according to the tumor environment [11,12]. Previous studies have also associated higher NLR with an unfavorable chemotherapy response and shorter survival in EOC [13,14]. Because LDH, CA125, and NLR provide partly different information on metabolism, tumor-associated antigen levels, and systemic inflammation, their combined assessment may provide complementary information beyond that obtained from a single marker.

In this study, we evaluated LDH, CA125, and NLR in patients with pathologically confirmed EOC and individually matched patients with benign ovarian lesions. We first examined the association between serum LDH and clinicopathological characteristics, and then assessed the diagnostic performance of the three markers alone and in continuous logistic regression models in the overall cohort and in early- and advanced-stage EOC. Separately, triple-positive status, defined by simultaneous elevation of LDH, CA125, and NLR above the prespecified cut-offs, was evaluated for its association with platinum resistance, progression-free survival (PFS), and overall survival (OS); an internally validated nomogram was also developed to estimate individual PFS probabilities.

## 2. Materials and Methods

### 2.1. Study Design and Ethical Approval

This study used a retrospective design that combined a 1:1 individually matched case–control analysis with a follow-up cohort analysis. The study was approved by the Ethics Committee of Beijing Chaoyang Hospital, Capital Medical University (protocol code 2026-sci-452; approval date: 16 April 2026), and the approval covered retrospective access to and analysis of clinical records and previously collected laboratory data from patients treated between 2015 and 2025. Written informed consent for the retrospective use of clinical data was obtained from all living participants; for patients who had died before consent was obtained, written authorization was obtained from their legally authorized representative.

### 2.2. Study Population

#### 2.2.1. Case–Control Study

Pathologically confirmed patients with primary EOC and patients with benign ovarian lesions treated at Beijing Chaoyang Hospital, Capital Medical University from January 2015 to January 2025 were included. Patients in the EOC group were required to have postoperative pathological confirmation of EOC, no previous antitumor treatment before the initial pretreatment laboratory assessment, and complete clinical, pathological, and laboratory data.

Benign controls were selected from patients who underwent surgery at the same institution during the same study period and had postoperative pathological confirmation of a benign ovarian lesion. They were required to meet the same pretreatment and data-availability criteria as the EOC group. Each EOC patient was individually matched to one benign control according to age (±3 years), BMI (±2 kg/m^2^), and menopausal status. Menopausal status was concordant in most matched pairs, although exact concordance was not achieved in two pairs. The histopathological composition of the benign ovarian lesion group is reported in Table 1.

Patients were excluded from either group if they had another primary malignancy; severe cardiac, hepatic, renal, or other major organ disease that could substantially affect serum LDH levels; hematological disorders such as hemolytic anemia, leukemia, or lymphoma; pregnancy or lactation; or severe psychiatric disease that prevented reliable data collection.

#### 2.2.2. The Cohort Study

The cohort study population was derived from the EOC patients enrolled in the aforementioned case–control study. Patients were included in the follow-up cohort if they met the EOC eligibility criteria, received first-line platinum-based chemotherapy, and had follow-up information available for assessment of disease progression and survival. Patients who discontinued first-line platinum-based chemotherapy for reasons unrelated to disease progression, such as severe adverse events or treatment refusal, were excluded from the platinum-resistance analysis. Adequate follow-up required for classification of platinum resistance is described in Section 2.6.

### 2.3. Data Collection

Demographic and clinical data were obtained from the electronic medical records, including age, menopausal status, BMI, smoking, chronic alcohol drinking, age at menarche, hormone replacement therapy, history of term delivery, and family history of cancer.

Tumor-related data included tumor distribution, pelvic mass size, pleural effusion, ascites, pelvic peritoneal dissemination, hepatic or splenic metastasis, vascular tumor thrombus, lymph-node metastasis, ascites cytology, histological subtype, pathological grade, and FIGO stage. Tumor distribution was divided into unilateral and non-unilateral disease, with the latter including bilateral tumors and diffuse lesions without a measurable dominant mass; histological subtype was grouped as serous or non-serous EOC for the regression analyses, and FIGO stages I–II and III–IV were classified as early and advanced disease, respectively.

Treatment data included the initial treatment strategy, lymph-node dissection, residual disease after surgery, first-line chemotherapy regimen, number and dates of chemotherapy cycles, and treatment modification. The initial treatment strategy was classified as primary debulking surgery (PDS) or neoadjuvant chemotherapy followed by interval debulking surgery (NACT–IDS). PDS was defined as cytoreductive surgery performed before first-line chemotherapy, whereas NACT–IDS referred to neoadjuvant chemotherapy followed by interval cytoreductive surgery. R0 resection was defined as no visible residual tumor after surgery.

### 2.4. Laboratory Measurements

Fasting peripheral venous blood was collected before surgery, chemotherapy, or other antitumor treatment. Serum LDH (ADVIA 2400 automated chemistry analyzer; Siemens Healthcare Diagnostics Inc., Tarrytown, NY, USA) and tumor markers, including CA125, CEA, CA19-9, and AFP (cobas e 601 analyzer; Roche Diagnostics GmbH, Mannheim, Germany), were measured within 7 days before the initial treatment, while routine blood tests (XN-20 automated hematology analyzer; Sysmex Corporation, Kobe, Japan) were performed within 3 days. All tests were completed in the clinical laboratory of Beijing Chaoyang Hospital according to standard procedures. NLR was calculated as the absolute neutrophil count divided by the absolute lymphocyte count, and all laboratory values were obtained from the original laboratory reports.

### 2.5. Biomarker Definitions

For the diagnostic analysis, LDH, CA125, and NLR were treated as continuous variables. Continuous biomarker values were entered into binary logistic regression models, and the predicted probabilities were used to construct ROC curves for the LDH+CA125, LDH+NLR, and LDH+CA125+NLR combined models. Separate logistic regression models were fitted for the overall, early-stage, and advanced-stage cohorts. For each combined model, the probability threshold used to calculate sensitivity and specificity was selected by maximizing the Youden index. Accordingly, the diagnostic AUC, sensitivity, and specificity reported for the three-marker combination refer exclusively to the continuous three-marker logistic regression model.

Triple-positive status was defined separately as a binary variable only for the platinum-resistance, PFS, and OS analyses. Patients were classified as triple-positive when all three biomarkers exceeded the study-specific cut-off values derived from the overall matched case–control cohort, namely LDH > 195.5 U/L, CA125 > 35.37 U/mL, and NLR > 2.63; all other patients were classified as non-triple-positive. These cut-offs were obtained from the overall matched case–control cohort and then applied consistently in the subsequent platinum-resistance, PFS, and OS analyses. Stage-specific cut-offs presented in the diagnostic analysis were used only to describe stage-specific single-marker diagnostic performance and were not used in the definition of triple-positive status.

### 2.6. Outcomes and Follow-Up

The primary outcome was progression-free survival (PFS), which was calculated from the date of the first treatment for EOC to the first disease progression, recurrence, or death from any cause. The first treatment date was the date of surgery or systemic antitumor treatment, whichever occurred first; patients without an event were censored at the date of their last confirmed disease assessment.

Disease progression was determined from clinical records and imaging findings, and measurable disease was assessed according to RECIST version 1.1. An isolated increase in CA125 without corresponding radiological or clinical evidence of disease progression was not considered a PFS event. When RECIST-measurable disease was absent, recurrence or progression was determined by unequivocal radiological or clinical evidence documented by the treating physician, including the appearance of new lesions or clear progression of non-measurable disease. The date of progression was defined as the date of the first imaging examination or clinical assessment that documented progression.

Secondary outcomes included platinum resistance and overall survival (OS). Platinum resistance was defined as progression during first-line platinum-based chemotherapy or within 6 months after the final platinum dose. Patients classified as non-platinum-resistant were required to have been followed for at least 6 months after completion of the final platinum-containing chemotherapy without evidence of progression during this period. OS was calculated from the first treatment date to death from any cause; patients who were alive at the data cut-off were censored at the last confirmed follow-up. Because no deaths occurred in patients with stage I–II disease, OS regression analysis was limited to patients with FIGO stage III-IV EOC.

Follow-up was performed through outpatient visits, hospital records, or telephone contact. Clinical examination, tumor-marker testing, and imaging were carried out according to routine clinical practice, and information on progression, recurrence, subsequent treatment, platinum response, and survival status was recorded. The follow-up data cut-off was 31 December 2025.

### 2.7. Variable Coding

Age was divided into ≤50 and >50 years, menopausal status into premenopausal and postmenopausal, tumor distribution into unilateral and non-unilateral, pathological subtype into non-serous and serous, pelvic mass size into <10 and ≥10 cm, histological subtype into low or moderate grade and high grade, and FIGO stage into stages I–II and III–IV. The initial treatment strategy was classified as PDS or NACT–IDS. Pleural effusion, ascites, pelvic peritoneal dissemination, hepatic or splenic metastasis, vascular tumor thrombus, lymph-node metastasis, and positive ascites cytology were coded as absent or present. Lymph-node dissection was coded as no or yes, while residual disease status was coded as non-R0 or R0; R0 indicated no visible residual tumor after surgery. Triple-positive status was coded as negative or positive.

### 2.8. Statistical Analysis

Continuous variables were tested for normality. Normally distributed data are presented as mean ± standard deviation, while skewed data are presented as median and interquartile range; categorical data are shown as number and percentage. In the 1:1 individually matched case–control analysis, paired-samples *t* tests were used for normally distributed continuous variables, Wilcoxon signed-rank tests for skewed variables, McNemar tests for binary variables, and marginal homogeneity tests for categorical variables with more than two groups. Within the EOC cohort, independent-samples *t* tests, Mann–Whitney U tests, χ^2^ tests, or Fisher’s exact tests were used as appropriate.

ROC curves were used to evaluate the diagnostic performance of individual biomarkers and combined logistic regression models. Continuous LDH, CA125, and NLR values were entered into binary logistic regression models, which were fitted separately for the overall, early-stage, and advanced-stage cohorts. AUCs with 95% confidence intervals, sensitivity, and specificity were calculated, and cut-off values or probability thresholds were selected using the maximum Youden index. Internal validation of the combined diagnostic models was performed using 1000 bootstrap resamples to obtain optimism-corrected AUCs. The incremental value of adding NLR to the LDH+CA125 model was assessed using the paired DeLong test and nested likelihood-ratio test.

To assess the incremental diagnostic value of NLR, the AUC of the LDH+CA125 model was compared with that of the LDH+CA125+NLR model using the paired DeLong test within the same complete-case cohort. Because a change in AUC may not fully reflect additional model information, nested likelihood-ratio tests were also used to evaluate whether adding NLR improved overall model fit.

Factors associated with platinum resistance were examined using univariable and multivariable logistic regression, and the results are reported as odds ratios (ORs) with 95% confidence intervals. This analysis was intended as a clinicopathological association analysis rather than a pretreatment prediction model. Variables for multivariable analysis were selected based on clinical relevance and the results of univariable analysis. Given the limited number of platinum-resistant events, the final multivariable analysis was restricted to three clinically relevant variables to reduce overfitting. When triple-positive status was included in the multivariable model, individual LDH, CA125, and NLR variables were not entered simultaneously because these biomarkers were components of the triple-positive definition.

Factors associated with PFS and OS were analyzed using univariable and multivariable Cox proportional-hazards regression, with results reported as hazard ratios (HRs) and 95% confidence intervals. The proportional-hazards assumption was checked for the final Cox models, and complete-case data were used for the variables included in each model. Kaplan–Meier curves were used to illustrate survival differences, with comparisons performed using the log-rank test and numbers at risk shown below the curves. A PFS nomogram was developed from factors retained in the multivariable Cox model. Because the nomogram included pathological grade, it was intended for postoperative prognostic assessment rather than pretreatment decision-making. Discrimination was assessed using Harrell’s C-index, calibration was evaluated at 12, 24, 36, 48, and 60 months, and internal validation was performed using 500 bootstrap resamples.

All analyses were performed using SPSS 26.0 (IBM Corp., Armonk, NY, USA), R 4.3.1 (R Foundation for Statistical Computing, Vienna, Austria), and GraphPad Prism 9 (GraphPad Software, San Diego, CA, USA); all tests were two-sided, and *p* < 0.05 was considered statistically significant.

## 3. Results

### 3.1. Patient Selection and Clinical Characteristics

**Figure 1:**
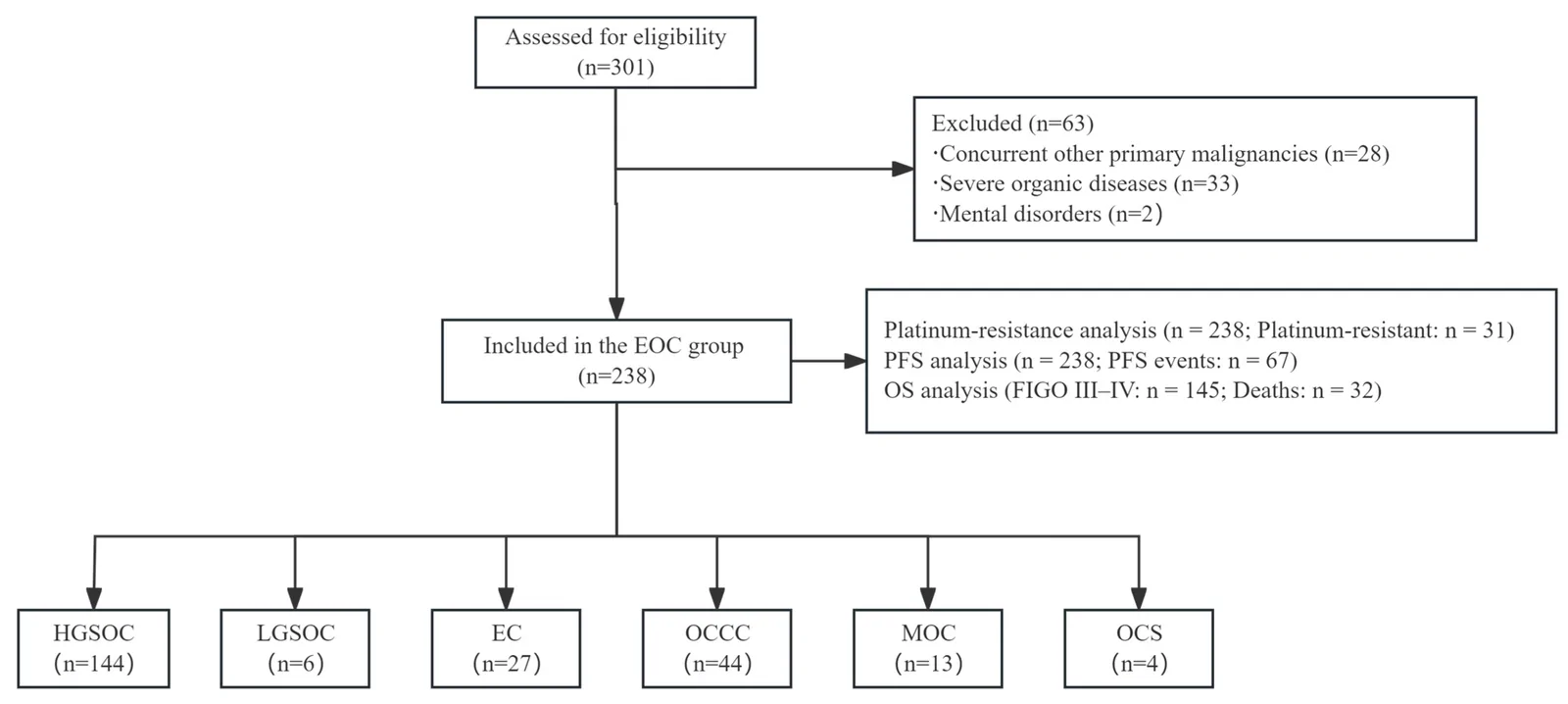
Flowchart of EOC patient selection, histological distribution, and analytic cohorts. EOC, epithelial ovarian cancer; PFS, progression-free survival; OS, overall survival; FIGO, International Federation of Gynecology and Obstetrics. HGSOC, high-grade serous ovarian carcinoma; LGSOC, low-grade serous ovarian carcinoma; EC, endometrioid carcinoma; OCCC, ovarian clear cell carcinoma; MOC, mucinous ovarian carcinoma; OCS, ovarian carcinosarcoma.

The patient-selection process is shown in Figure 1. Among the 301 patients with EOC who were assessed for eligibility, 63 were excluded, including 28 with another primary malignancy, 33 with severe major organ disease, and 2 with severe psychiatric disorders. Finally, 238 patients with EOC were included and individually matched 1:1 with 238 patients with benign ovarian lesions according to age, menopausal status, and BMI. The histopathological distributions of both groups are summarized in Table 1. Serous cystadenoma was the most common benign ovarian lesion, accounting for 83 cases (34.87%). High-grade serous carcinoma was the predominant EOC histological subtype, accounting for 144 cases (60.50%). The FIGO stage distribution varied across histological subtypes. Most patients with high-grade serous carcinoma had advanced-stage disease (116/144, 80.56%), whereas early-stage disease was more frequent in endometrioid carcinoma (23/27, 85.19%), mucinous carcinoma (10/13, 76.92%), and clear cell carcinoma (27/44, 61.36%). Overall, 92 patients (38.66%) had FIGO stage I–II disease and 146 (61.34%) had FIGO stage III–IV disease. The detailed FIGO stage distribution for each histological subtype is presented in Table 1.

After 1:1 individual matching, age, menopausal status, BMI, smoking history, chronic alcohol drinking, and hormone replacement therapy history were similar between the two groups (all *p* > 0.05). Compared with patients with benign ovarian lesions, patients with EOC had a later age at menarche, a lower proportion with a history of term delivery, and a higher proportion with a family history of cancer (all *p* < 0.05). Unilateral tumors were less common in the EOC group, whereas bilateral tumors and diffuse lesions were more frequent; patients with EOC also had larger pelvic masses and higher rates of pleural effusion and ascites (all *p* < 0.05). Serum LDH, NLR, and CA125 levels were higher in the EOC group than in the benign ovarian lesion group (all *p* < 0.0001), while no significant differences were found in CEA, CA19-9, or AFP levels. The detailed clinical and laboratory characteristics are shown in Table 2.

### 3.2. Comparison of LDH Levels Across Clinicopathological Characteristics

**Figure 2:**
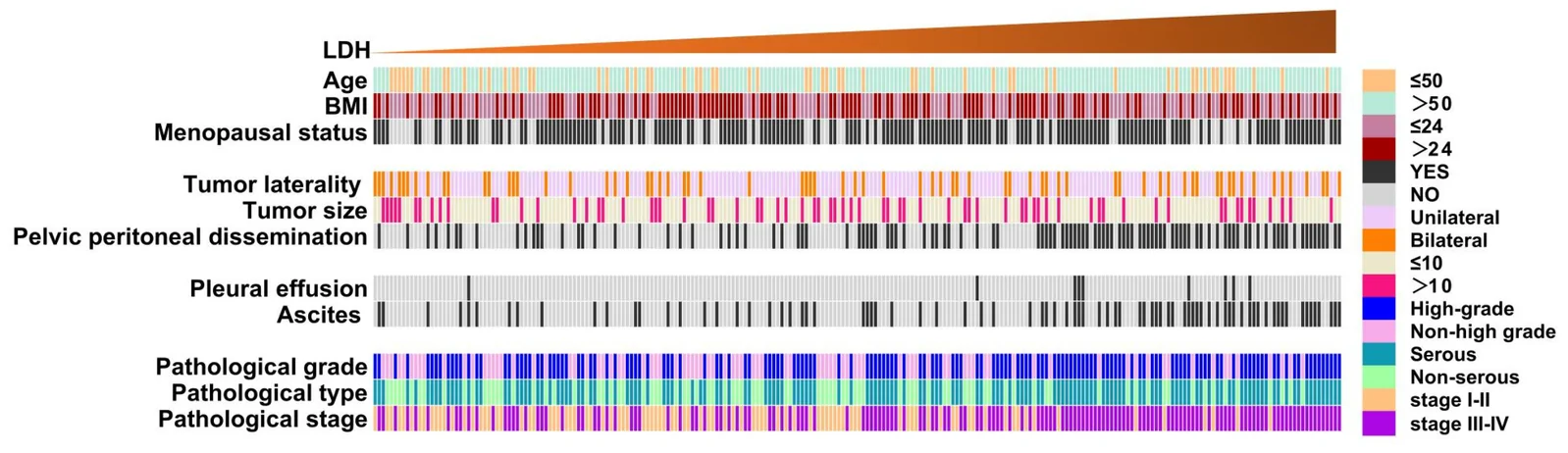
Clinicopathological characteristics of patients with EOC. Each column represents an individual patient, and rows represent clinicopathological variables. Patients were ordered by serum LDH level from left to right in ascending order, with the gradient bar at the top indicating increasing LDH concentrations across the cohort.

Serum LDH levels did not differ significantly according to age, BMI, menopausal status, tumor distribution, or pelvic mass size in patients with EOC; similarly, no significant differences were observed according to hepatic metastasis, splenic metastasis, pleural effusion, or lymph-node metastasis (all *p* > 0.05). In contrast, LDH levels were higher in patients with pelvic peritoneal dissemination, ascites, positive ascites cytology, or vascular tumor thrombus than in those without these features, and patients with ascites had the highest LDH levels. Higher LDH levels were also found in patients with serous EOC, high pathological grade, and FIGO stage III–IV disease (all *p* < 0.05). The distribution of clinicopathological characteristics according to serum LDH level is shown in Figure 2, and the detailed comparisons are presented in Table 3.

### 3.3. Diagnostic Performance of LDH, CA125, NLR, and Their Combined Models for EOC

**Figure 3:**
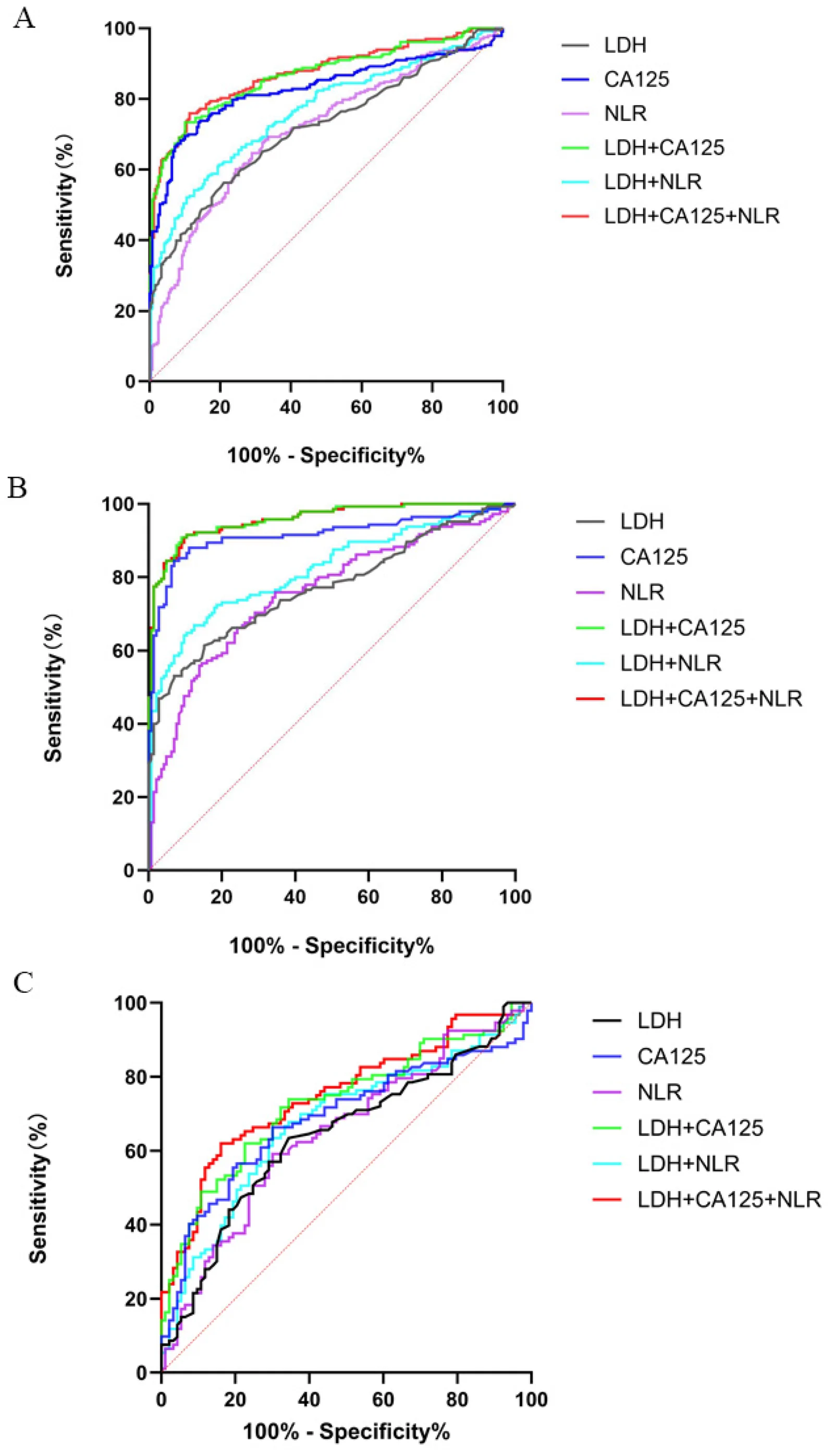
ROC curves for LDH, CA125, NLR, and combined logistic regression models in the overall, early-stage, and advanced-stage EOC cohorts. (**A**) Total cohort; (**B**) advanced-stage EOC, FIGO stages III–IV; (**C**) early-stage EOC, FIGO stages I–II.

The diagnostic performance of LDH, CA125, and NLR was assessed in the total cohort, and separate analyses were also carried out for early- and advanced-stage EOC. The ROC curves are shown in Figure 3, while the corresponding diagnostic parameters are listed in Table 4. In the total cohort, CA125 showed the highest AUC among the single markers, with an AUC of 0.829 (95% CI: 0.789–0.868). LDH and NLR had similar diagnostic performance, with AUCs of 0.717 and 0.711, respectively. Among the continuous combined models, the LDH+CA125+NLR model had the highest AUC of 0.873 (95% CI: 0.840–0.905), with a sensitivity of 75.97% and a specificity of 88.66%. The LDH+CA125 model had a similar AUC of 0.867, a sensitivity of 73.39%, and a specificity of 89.92%.

Diagnostic performance was higher in advanced-stage EOC than in early-stage disease. In patients with FIGO stage III–IV EOC, CA125 alone had an AUC of 0.915, with a sensitivity of 84.40% and a specificity of 93.10%. Both the LDH+CA125 and LDH+CA125+NLR models had an AUC of 0.958; their sensitivities were 90.85% and 91.55%, and their specificities were 91.03% and 89.66%, respectively. In patients with FIGO stage I–II EOC, the AUCs of all single markers were below 0.70. The LDH+CA125+NLR model had an AUC of 0.751 (95% CI: 0.680–0.821), with a sensitivity of 61.96% and a specificity of 83.87%.

Because the distribution of FIGO stage differed across histological subtypes, a sensitivity analysis was performed to assess whether histological composition could account for the better diagnostic performance observed in advanced-stage EOC. Patients were stratified into HGSOC and non-HGSOC groups, and diagnostic performance was evaluated separately in early- and advanced-stage disease (Table 5). Within the HGSOC group, the AUC of the LDH+CA125+NLR model increased from 0.809 (95% CI: 0.713–0.905) in FIGO stage I–II disease to 0.969 (95% CI: 0.950–0.987) in FIGO stage III–IV disease. A similar pattern was observed in the non-HGSOC group, with the corresponding AUC increasing from 0.697 (95% CI: 0.619–0.775) to 0.936 (95% CI: 0.891–0.981). The similar stage-related pattern in both histological groups indicates that the higher diagnostic performance in advanced-stage disease was unlikely to be explained solely by histological composition.

The specifications and internal validation results of the combined diagnostic models are shown in Table 6. Bootstrap correction resulted in only small changes in AUC, with corrected AUCs of 0.870, 0.738, and 0.954 for the LDH+CA125+NLR model in the overall, early-stage, and advanced-stage cohorts, respectively. Adding NLR to the LDH+CA125 model did not significantly improve the AUC in the overall (DeLong *p* = 0.092), early-stage (*p* = 0.157), or advanced-stage (*p* = 0.778) cohorts. However, the likelihood-ratio test showed improved model fit after adding NLR in the early-stage cohort (*p* = 0.019), whereas no significant improvement was observed in the overall or advanced-stage cohorts.

### 3.4. Factors Associated with Platinum Resistance in EOC

All 238 patients with EOC had sufficient follow-up for platinum-resistance classification; 31 were classified as platinum-resistant and 207 as non-platinum-resistant, with all non-resistant patients having at least 6 months of progression-free follow-up after the final platinum-containing chemotherapy. In the univariable logistic regression, higher LDH (OR = 3.023, 95% CI: 1.248–7.326; *p* = 0.014), triple-positive status (OR = 4.455, 95% CI: 2.012–9.861; *p* < 0.001), ascites (OR = 3.703, 95% CI: 1.695–8.090; *p* = 0.001), positive ascites cytology (OR = 2.596, 95% CI: 1.204–5.599; *p* = 0.015), and FIGO stage III–IV disease (OR = 3.021, 95% CI: 1.189–7.678; *p* = 0.020) were associated with higher odds of platinum resistance. CA125 and NLR alone were not significantly associated with platinum resistance. Primary debulking surgery was also not significantly associated with platinum resistance (OR = 2.651, 95% CI: 0.603–11.667; *p* = 0.197). In the multivariable analysis, triple-positive status remained significantly associated with platinum resistance (adjusted OR = 3.488, 95% CI: 1.504–8.087; *p* = 0.004), whereas ascites (adjusted OR = 2.697, 95% CI: 0.986–7.379; *p* = 0.053) and FIGO stage III–IV disease (adjusted OR = 1.057, 95% CI: 0.311–3.593; *p* = 0.930) were not statistically significant (Table 7).

### 3.5. Analysis of Prognostic Factors for PFS and OS in EOC

Survival analyses included all 238 patients with EOC, among whom 67 experienced progression, recurrence, or death during follow-up. The median observed follow-up duration was 32.7 months (interquartile range, 20.2–57.0 months; range, 5.7–107.8 months). The OS analysis was restricted to 145 patients with FIGO stage III–IV disease, among whom 32 deaths occurred. In this subgroup, the median observed follow-up duration was 31.8 months (interquartile range, 17.9–51.5 months; range, 5.7–107.8 months).

**Figure 4:**
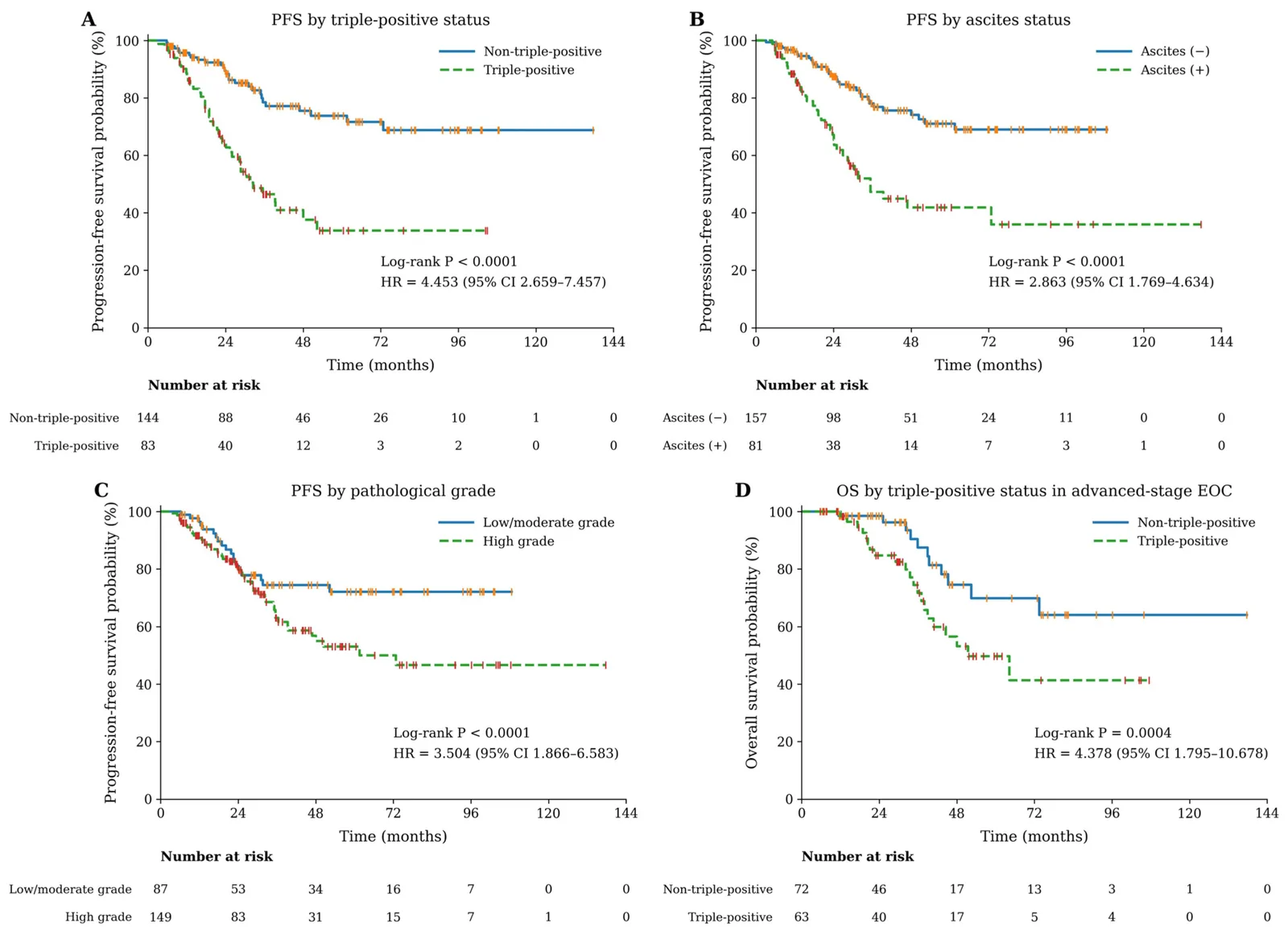
Kaplan–Meier survival curves for PFS and OS. (**A**) PFS according to triple-positive status. (**B**) PFS according to ascites status. (**C**) PFS according to pathological grade. (**D**) OS according to triple-positive status in patients with advanced-stage EOC. Numbers at risk are shown below each curve. Red and orange markers indicate censored data.

Univariable Cox regression identified CA125, triple-positive status, tumor distribution, ascites, pathological grade, histological subtype, and FIGO stage as factors associated with PFS. In the multivariable model, triple-positive status remained independently associated with an increased risk of progression or death (HR = 3.109, 95% CI: 1.772–5.456, *p* < 0.001). Ascites (HR = 2.208, 95% CI: 1.181–4.126, *p* = 0.013) and high pathological grade (HR = 2.235, 95% CI: 1.075–4.646, *p* = 0.031) were also independent adverse prognostic factors for PFS, whereas tumor distribution and FIGO stage were no longer significant after adjustment (Table 8). Kaplan–Meier analysis further illustrated the prognostic differences associated with these variables (Figure 4A–C). Patients with triple-positive status had markedly shorter PFS than those without triple positivity (Log-rank *p* < 0.0001); ascites and high pathological grade were also associated with shorter PFS (both log-rank *p* < 0.0001).

For OS, in the advanced-stage cohort, univariable Cox regression showed that triple-positive status (HR = 4.378, 95% CI: 1.795–10.678; *p* = 0.001), pelvic mass size ≥ 10 cm (HR = 2.221, 95% CI: 1.095–4.505; *p* = 0.027), and ascites (HR = 2.328, 95% CI: 1.075–5.040; *p* = 0.032) were associated with shorter OS. In the multivariable analysis, triple-positive status remained independently associated with mortality (HR = 3.538, 95% CI: 1.385–9.038; *p* = 0.008), whereas pelvic mass size ≥ 10 cm (HR = 1.350, 95% CI: 0.643–2.835; *p* = 0.428) and ascites (HR = 1.988, 95% CI: 0.906–4.360; *p* = 0.086) were not statistically significant (Table 9). Kaplan–Meier analysis also showed shorter OS in patients with triple-positive status (Figure 4D; log-rank *p* = 0.0004).

**Figure 5:**
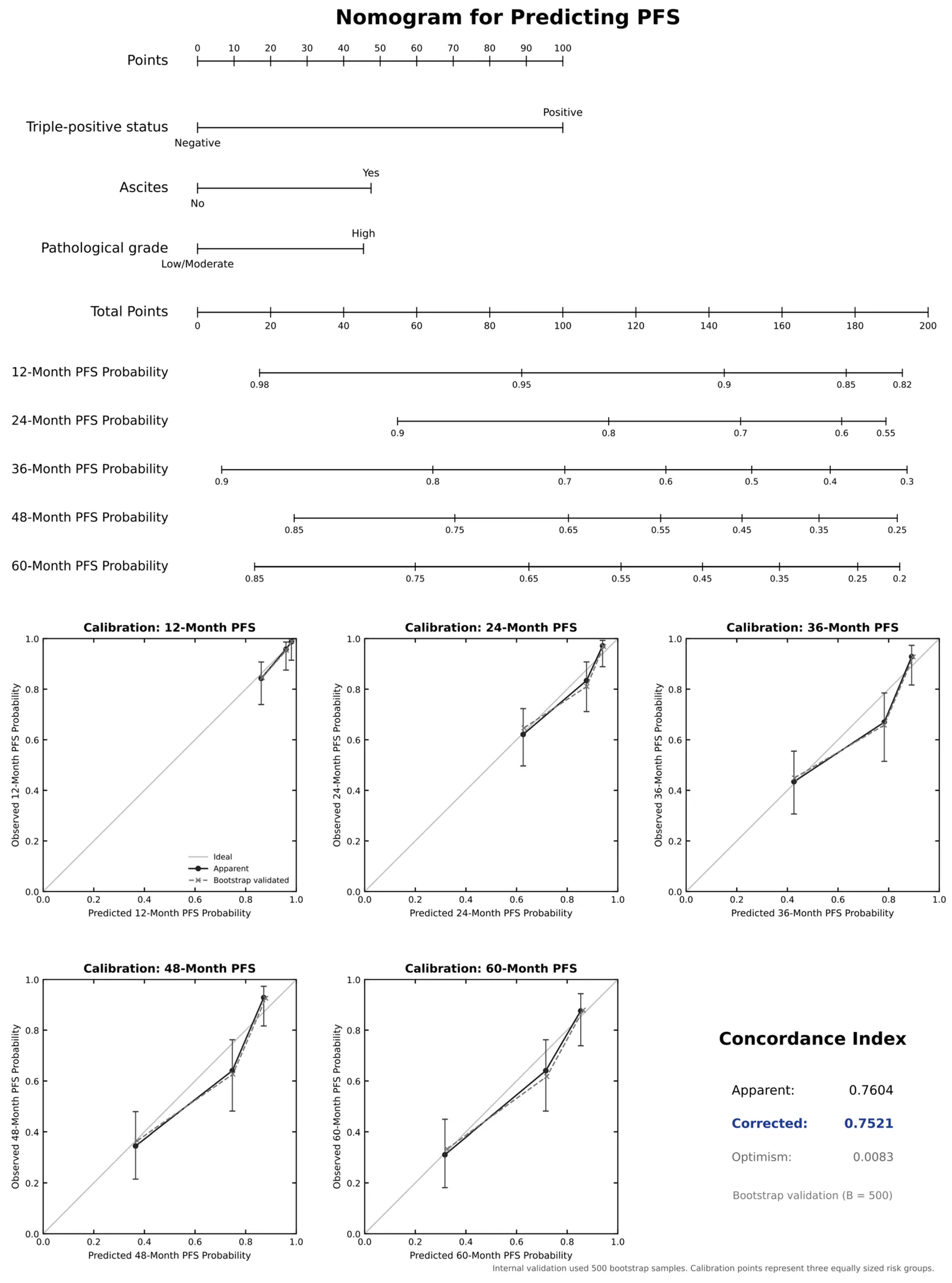
Nomogram for predicting PFS in patients with epithelial ovarian cancer and its internal validation. The upper panel shows the prognostic nomogram incorporating triple-positive status, ascites, and pathological grade to estimate 12-, 24-, 36-, 48-, and 60-month PFS probabilities. The lower panel presents calibration curves for the five prespecified time points and the concordance index of the model.

Based on the three independent factors associated with PFS, namely triple-positive status, ascites, and pathological grade, a prognostic nomogram was developed to estimate 12-, 24-, 36-, 48-, and 60-month PFS probabilities (Figure 5). The model had an apparent C-index of 0.7604; after 500 bootstrap resamples, the corrected C-index was 0.7521, with an optimism estimate of 0.0083. The calibration curves showed generally close agreement between the predicted and observed PFS probabilities across the five time points.

## 4. Discussion

### 4.1. Diagnostic Value of Serum Biomarkers in EOC

Despite the widespread use of serum biomarkers, the diagnosis of EOC remains difficult because currently available markers do not show sufficient sensitivity and specificity in all patients. CA125 is a classic marker for EOC, but its sensitivity is relatively low in early-stage disease; elevated CA125 levels may also occur in endometriosis, menstruation, pregnancy, inflammation, and other benign conditions [4,15]. NLR reflects tumor-related systemic inflammation, but its prognostic associations may vary according to tumor type and clinical setting [11,14]. In the present study, serum LDH, CA125, and NLR levels were significantly higher in patients with EOC than in those with benign ovarian lesions. LDH was also associated with pelvic peritoneal dissemination, ascites, positive ascites cytology, vascular tumor thrombus, serous pathological type, high pathological grade, and advanced FIGO stage. These findings suggest that serum LDH may partly reflect tumor burden and aggressive disease behavior, although it should not be regarded as an ovarian cancer-specific marker.

The continuous LDH+CA125+NLR model showed higher diagnostic performance in advanced-stage than in early-stage EOC. Although HGSOC was more frequently diagnosed at advanced stages, the same stage-related pattern was observed in both the HGSOC and non-HGSOC groups, suggesting that histological composition alone did not explain this difference. Greater tumor burden and more pronounced metabolic and inflammatory changes in advanced disease may partly contribute to the higher diagnostic performance [11,14]. However, the small numbers of patients in some histological subtypes limited further subgroup analyses.

Internal validation showed only small changes in model performance after bootstrap correction. Importantly, adding NLR to LDH+CA125 did not significantly improve the AUC in any stage group, indicating that the numerically higher AUC of the three-marker model should not be interpreted as clear superiority. In early-stage EOC, adding NLR improved overall model fit and increased specificity, although sensitivity decreased. NLR may therefore provide complementary information in early-stage disease, particularly through improved overall model fit, although this finding still requires external validation. The three indicators reflect related but different changes. CA125 mainly reflects MUC16-associated tumor activity, LDH is related to tumor metabolism and tissue turnover, while NLR provides information on the systemic inflammatory and immune state. Their combined use may therefore compensate for some of the limitations of a single marker. The panel is also based on routine blood tests and does not require an additional testing platform. Recent studies have continued to evaluate CA125, HE4, ROMA, RMI, ultrasound models, and other biomarker combinations for the assessment of ovarian masses [16,17,18]. Comparative studies have also shown that established biomarker- and ultrasound-based approaches may differ considerably in both discrimination and clinical utility, emphasizing the need to evaluate new blood-based models against these established methods rather than on AUC alone [19]. However, these methods were not directly compared with the LDH+CA125+NLR panel in the same patients; therefore, the current findings cannot demonstrate superiority or non-inferiority to HE4-, ROMA-, RMI-, or ultrasound-based approaches. The continuous three-marker model should therefore currently be considered a supplementary blood-based approach rather than a replacement for established diagnostic methods.

The diagnostic findings should also be interpreted according to the design of this study. Patients with pathologically confirmed EOC were compared with individually matched patients with benign ovarian lesions who underwent surgery at the same institution. The results therefore describe discrimination between EOC and benign ovarian lesions within a selected surgical population, rather than performance in asymptomatic women or in population screening. External validation in broader and less selected populations of patients with adnexal masses, together with direct comparison with established imaging and biomarker-based approaches, will be needed to determine the generalizability and clinical usefulness of the model.

### 4.2. Association of the Triple-Positive Status with Platinum Resistance

Platinum-based chemotherapy remains the main first-line systemic treatment for EOC, but platinum resistance is still an important reason for recurrence and treatment failure. The development of resistance involves several processes, including altered drug transport, increased DNA damage repair, changes in apoptosis, metabolic adaptation, tumor-cell plasticity, and changes in the immune microenvironment [20,21,22,23,24]. The heterogeneous nature of these mechanisms may partly explain why a single circulating marker cannot fully reflect platinum responsiveness.

In the univariable analysis, elevated LDH and triple-positive status were associated with platinum resistance, whereas CA125 and NLR alone did not reach statistical significance. Ascites, positive ascites cytology, and advanced FIGO stage were also associated with platinum resistance, suggesting that the observed biomarker pattern may be related, at least partly, to more extensive disease. A previous ovarian cancer study reported an association between pretreatment LDH, platinum resistance, and survival [10]. The significant association of LDH in the present analysis is broadly consistent with that observation, although circulating LDH is influenced by tumor burden and other systemic conditions and cannot be regarded as a specific marker of drug resistance. In the final multivariable model, triple-positive status remained associated with platinum resistance after adjustment for ascites and FIGO stage. This finding suggests that the simultaneous elevation of LDH, CA125, and NLR may reflect a clinical phenotype that is not completely captured by disease stage or ascites alone. However, the present analysis demonstrates an association rather than a validated ability to predict platinum resistance.

Several biological findings may partly explain this association. Metabolic changes and increased lactate production have been linked to tumor-cell adaptation and reduced treatment sensitivity [7,9,23]. CA125 is derived from MUC16, a transmembrane mucin involved in tumor-cell adhesion, peritoneal dissemination, and immune regulation [25]. Experimental evidence has also shown that MUC16 may stimulate neutrophils toward an inflammatory and immunosuppressive state, which provides a possible link between CA125-related tumor activity and systemic inflammatory changes [26,27]. Together, these observations provide a possible explanation for why simultaneous abnormalities in LDH, CA125, and NLR may be associated with resistant disease. However, these mechanisms were not directly examined in our cohort, and triple-positive status should therefore be interpreted as a clinical association rather than a causal mechanism of platinum resistance.

The confidence interval for the adjusted OR was relatively wide, and the limited number of platinum-resistant events reduced the precision of the estimate. PDS was not significantly associated with platinum resistance in the present cohort, although this finding should be interpreted cautiously because treatment strategy is closely related to disease extent, resectability, and patient characteristics. These issues, together with other potential sources of residual confounding, are discussed further in Section 4.4.

### 4.3. Prognostic Significance of the Triple-Positive Panel in EOC

The updated survival analysis identified triple-positive status, ascites, and high pathological grade as factors independently associated with shorter PFS. Among these factors, triple-positive status showed a consistent association with unfavorable outcome in both the Cox regression and Kaplan–Meier analyses. These findings suggest that the combined serum pattern may capture prognostic information that is not fully represented by conventional clinicopathological characteristics alone. PDS was not significantly associated with PFS in the present cohort, although treatment strategy is closely related to disease extent, resectability, and patient selection and should therefore not be interpreted as having no prognostic relevance.

Ascites is common in advanced EOC and is not simply a passive collection of fluid. It contains free tumor cells, multicellular aggregates, immune cells, stromal cells, cytokines, extracellular vesicles, and other soluble components; these elements may contribute to peritoneal implantation, immune suppression, and reduced treatment sensitivity [28,29]. Single-cell analysis has also shown that malignant ascites is connected with the cellular and immune environments of primary and metastatic ovarian tumors [28]. The independent association between ascites and PFS in the present study is therefore consistent with its clinical and biological characteristics. Ascites was associated with OS in the univariable analysis, but this association was attenuated after multivariable adjustment. This may reflect overlap between ascites and other manifestations of advanced intraperitoneal disease rather than an absence of prognostic relevance.

High pathological grade was another independent adverse factor for PFS. High-grade tumors are generally less differentiated and show more aggressive clinical behavior, although the effect of grade differs among ovarian cancer histological subtypes. Previous studies have also shown that stage, histotype, and grade remain important factors associated with ovarian cancer survival [30,31]. In the present analysis, pathological grade remained significant after adjustment for triple-positive status, ascites, tumor distribution, and FIGO stage, which is consistent with the established prognostic importance of tumor biology in EOC.

For OS, the analysis was limited to patients with advanced-stage EOC, and triple-positive status remained associated with mortality after multivariable adjustment. A similar unfavorable association was therefore observed for both PFS and OS, although the two analyses involved different study populations and should not be interpreted as identical prognostic models. The three components of triple-positive status reflect different aspects of EOC: LDH is associated with metabolic activity and tissue turnover, CA125 reflects MUC16-associated tumor activity and burden, while NLR reflects the systemic inflammatory response [7,8,9,25,26,27]. Their simultaneous elevation may therefore represent the coexistence of greater metabolic activity, tumor burden, and systemic inflammation, providing a possible biological explanation for its association with unfavorable survival. These mechanisms were not directly examined in the present study, and the observed association should not be interpreted as evidence of causality.

A PFS nomogram was constructed using triple-positive status, ascites, and pathological grade. Its bootstrap-corrected C-index of approximately 0.75 indicated moderate discrimination, while the calibration analysis showed generally close agreement between predicted and observed PFS probabilities within the study cohort. Previous ovarian cancer nomograms have also combined circulating markers with clinical or pathological factors to estimate recurrence and survival [32,33]. The small difference between the apparent and bootstrap-corrected C-index suggests limited optimism within the present dataset, but internal validation cannot establish performance in an independent population. Current guidance recommends that clinical prediction models should report discrimination, calibration, internal validation, and subsequent external validation clearly [34,35]. External validation will therefore be important to determine the reproducibility and potential clinical applicability of this nomogram.

### 4.4. Limitations

This study has several limitations. First, its retrospective single-center design may have introduced selection bias, and the diagnostic analysis was based on a selected surgical population, which may limit generalizability. Second, the numbers of platinum-resistant cases and deaths were relatively small, reducing the precision of the corresponding regression estimates. Third, LDH, CA125, and NLR were measured only before treatment, and their dynamic changes were not evaluated. BRCA1/2 and HRD data were not systematically available, while maintenance treatment and subsequent treatment after recurrence were not fully considered, which may have resulted in residual confounding [36,37]. In addition, the diagnostic models and PFS nomogram received only internal bootstrap validation and require external validation. Finally, this study identified clinical associations but did not directly investigate the underlying biological mechanisms.

## 5. Conclusions

In this study, the continuous logistic regression model combining LDH, CA125, and NLR showed diagnostic discrimination for distinguishing EOC from benign ovarian lesions, particularly in advanced-stage disease, although adding NLR did not significantly improve the AUC over LDH+CA125. Separately, triple-positive status was independently associated with platinum resistance, shorter PFS in the overall EOC cohort, and shorter OS in patients with advanced-stage EOC. The PFS nomogram constructed using triple-positive status, ascites, and pathological grade showed moderate discrimination and acceptable calibration after internal validation. These findings support further evaluation of routine blood-based markers for diagnostic and prognostic assessment in EOC.

## Figures and Tables

**Table 1:** Histopathological distribution of benign ovarian lesions and epithelial ovarian cancers.

**Panel A. Histopathological Distribution of Benign Ovarian Lesions**							
Benign ovarian lesions	*n* (%)						
Serous cystadenoma	83 (34.87)						
Mature teratoma	33 (13.87)						
Mucinous cystadenoma	26 (10.92)						
Endometriotic cyst	26 (10.92)						
Inclusion cyst	23 (9.66)						
Functional/simple cysts	18 (7.56)						
Mesonephric/paramesonephric cysts	14 (5.88)						
Fibroma/thecoma	7 (2.94)						
Serous cystadenofibroma	2 (0.84)						
Other benign lesions	6 (2.52)						
**Panel B. FIGO stage distribution according to histological subtype in epithelial ovarian cancer**							
**Histological subtype**	**Total** ***n* (%)**	**FIGO I** ***n* (%)**	**FIGO II** ***n* (%)**	**FIGO III** ***n* (%)**	**FIGO IV** ***n* (%)**	**Early I–II** ***n* (%)**	**Advanced III–IV** ***n* (%)**
High-grade serous carcinoma	144 (60.50)	13 (9.03)	15 (10.42)	92 (63.89)	24 (16.67)	28 (19.44)	116 (80.56)
Low-grade serous carcinoma	6 (2.52)	1 (16.67)	1 (16.67)	3 (50.00)	1 (16.67)	2 (33.33)	4 (66.67)
Endometrioid carcinoma	27 (11.34)	17 (62.96)	6 (22.22)	4 (14.81)	0 (0.00)	23 (85.19)	4 (14.81)
Clear cell carcinoma	44 (18.49)	22 (50.00)	5 (11.36)	14 (31.82)	3 (6.82)	27 (61.36)	17 (38.64)
Mucinous carcinoma	13 (5.46)	9 (69.23)	1 (7.69)	3 (23.08)	0 (0.00)	10 (76.92)	3 (23.08)
Ovarian carcinosarcoma	4 (1.68)	0 (0.00)	2 (50.00)	2 (50.00)	0 (0.00)	2 (50.00)	2 (50.00)

Note: Percentages in Panel A are calculated using the benign ovarian lesion cohort (*n* = 238) as the denominator. In Panel B, percentages for each FIGO stage are calculated within each histological subtype, while percentages in the total row are calculated using the entire EOC cohort (*n* = 238). Early-stage disease was defined as FIGO stages I–II and advanced-stage disease as FIGO stages III–IV. Functional/simple cysts included simple cysts (*n* = 8), follicular cysts (*n* = 8), and corpus luteum cysts (*n* = 2). Other benign lesions included struma ovarii, theca-cell luteinization, mesosalpinx cyst, unspecified cystadenoma, unspecified ovarian cyst, and ovarian corpus albicans (*n* = 1 each).

**Table 2:** Comparison of clinical characteristics between EOC and benign ovarian lesion groups.

Characteristic	Benign Ovarian Lesion Group (n = 238)	EOC Group (n = 238)	p-Value
**Demographics**			
Age, years	57.27 ± 11.82	57.88 ± 11.81	0.577
Menopausal status			
Yes	170 (71.43%)	172 (72.27%)	0.839
No	68 (28.57%)	66 (27.73%)	
BMI, kg/m^2^	24.51 ± 3.24	24.19 ± 3.60	0.307
History of smoking			
Yes	4 (1.68%)	6 (2.52%)	0.749
No	234 (98.32%)	232 (97.48%)	
History of chronic alcohol drinking			
Yes	2	0 (0)	0.498
No	236	238 (100.00%)	
Age at menarche, years	13.11 ± 0.98	13.71 ± 1.46	<0.0001
HRT			
Yes	1 (0.42%)	3 (1.24%)	0.625
No	237 (99.58%)	235 (98.76%)	
History of term delivery			
Yes	215 (90.34%)	200 (84.03%)	0.040
No	23 (9.66%)	38 (15.97%)	
Family history of cancer			
Yes	18 (7.56%)	47 (19.75%)	0.0001
No	220 (92.44%)	191 (80.25%)	
**Tumor characteristics**			
Tumor laterality			
Unilateral	197 (82.77%)	155 (65.13%)	<0.0001
Bilateral	41 (17.23%)	71 (29.83%)	
Diffuse lesions (no measurable tumor)	0 (0)	12 (5.04%)	
Pelvic mass size, cm	6.58 ± 3.76	9.02 ± 6.55	<0.0001
Pleural effusion			
Yes	1 (0.42%)	9 (3.78%)	0.025
No	237 (99.58%)	229 (96.22%)	
Ascites			
Yes	6 (2.52%)	81 (34.03%)	<0.0001
No	232 (97.48%)	157 (65.97%)	
**Laboratory parameters**			
LDH, U/L	174.20 ± 27.90	224.20 ± 90.97	<0.0001
NLR	2.05 (1.51, 2.82)	3.28(2.10, 5.17)	<0.0001
CA125, U/mL	13.20 (8.37, 21.46)	142.20 (31.15, 660.80)	<0.0001
CEA, ng/mL	1.20 (0.60, 1.83)	0.92 (0.40, 1.82)	0.107
CA19-9, U/mL	12.98 (7.79, 24.58)	12.91 (6.25, 30.07)	0.695
AFP, ng/mL	2.62 (2.00, 3.80)	2.70 (1.70, 3.94)	0.928

**Table 3:** Comparison of serum LDH levels according to clinicopathological characteristics in patients with EOC.

Index	LDH	p-Value
Age, years		
≤50	207.50 ± 67.37	0.053
>50	229.60 ± 97.67	
BMI		
<18.5	229.90 ± 104.8	0.548
18.5 ≤ BMI < 24	214.60 ± 109.7	
24 ≤ BMI < 28	213.40 ± 63.48	
≥28	234.10 ± 102.5	
Menopausal status		
Yes	229.60 ± 98.17	0.071
No	209.10 ± 68.56	
Tumor laterality		
Unilateral	220.30 ± 76.63	0.432
Non-unilateral	232.70 ± 121.40	
Pelvic mass size, cm		
<10	227.40 ± 97.84	0.303
≥10	215.50 ± 72.99	
Pelvic peritoneal dissemination		
Yes	254.40 ± 110.9	<0.0001
No	198.10 ± 59.76	
Hepatic metastasis		
Yes	245.60 ± 66.49	0.191
No	222.20 ± 92.79	
Splenic metastasis		
Yes	205.90 ± 81.52	0.545
No	224.50 ± 91.67	
Pleural effusion		
Yes	257.10 ± 55.14	0.106
No	222.60 ± 92.21	
Ascites		
Yes	264.40 ± 122.40	<0.0001
No	203.00 ± 60.68	
Histological subtype		
Serous	235.80 ± 104.30	0.003
Non-serous	203.60 ± 58.27	
Pathological grade		
Low/moderate	202.10 ± 58.87	0.001
High	236.90 ± 104.00	
Vascular tumor thrombus		
Yes	244.30 ± 108.60	0.0001
No	201.00 ± 59.10	
Lymph node metastasis		
Yes	217.10 ± 83.17	0.573
No	225.30 ± 92.98	
Positive ascites cytology		
Yes	256.60 ± 116.70	0.0001
No	204.00 ± 64.23	
FIGO stage		
I–II	187.20 ± 39.80	<0.0001
III–IV	247.50 ± 106.10	

**Table 4:** Diagnostic performance parameters of LDH, CA125, NLR and their combined models in EOC patients.

Index	Cohort	AUC (95%CI)	Sensitivity (%) (95%CI)	Specificity (%) (95%CI)	YI	Cut-Off Value
LDH	Total	0.717 (0.671–0.763)	56.30% (0.500–0.625)	78.99% (0.734–0.837)	0.35	195.5 U/L
	Stage I–II	0.640 (0.560–0.721)	63.44% (0.533–0.725)	65.59% (0.555–0.745)	0.29	177.50 U/L
	Stage III–IV	0.772 (0.717–0.826)	61.38% (0.533–0.689)	84.83% (0.781–0.898)	0.46	202.5 U/L
CA125	Total	0.829 (0.789–0.868)	73.82% (0.678–0.791)	85.71% (0.807–0.896)	0.60	35.37 U/mL
	Stage I–II	0.685 (0.606–0.764)	66.30% (0.562–0.751)	68.89% (0.599–0.783)	0.36	30.25 U/mL
	Stage III–IV	0.915 (0.878–0.952)	84.40% (0.775–0.895)	93.1% (0.878–0.962)	0.78	52.05 U/mL
NLR	Total	0.711 (0.665–0.757)	64.71% (0.584–0.705)	71.01% (0.650–0.764)	0.36	2.63
	Stage I–II	0.645 (0.566–0.724)	59.14% (0.490–0.686)	69.89% (0.599–0.783)	0.29	2.48
	Stage III–IV	0.756 (0.700–0.812)	55.86% (0.477–0.637)	86.21% (0.797–0.909)	0.42	3.32
LDH+CA125	Total	0.867 (0.834–0.901)	73.39% (0.674–0.787)	89.92% (0.854–0.931)	0.63	-
	Stage I–II	0.727 (0.654–0.801)	73.91% (0.641–0.818)	65.59% (0.555–0.745)	0.40	-
	Stage III–IV	0.958 (0.937–0.979)	90.85% (0.850–0.923)	91.03% (0.853–0.947)	0.82	-
LDH+NLR	Total	0.759 (0.716–0.803)	51.93% (0.455–0.583)	89.50% (0.850–0.928)	0.41	-
	Stage I–II	0.673 (0.597 to 0.750)	67.39% (0.573–0.761)	66.67% (0.566–0.754)	0.34	-
	Stage III–IV	0.817 (0.768–0.867)	64.08% (0.559–0.715)	90.34% (0.844–0.942)	0.54	-
LDH+CA125+NLR	Total	0.873 (0.840–0.905)	75.97% (0.701–0.810)	88.66% (0.840–0.921)	0.65	-
	Stage I–II	0.751 (0.680–0.821)	61.96% (0.518–0.712)	83.87% (0.751–0.900)	0.46	-
	Stage III–IV	0.958 (0.937–0.979)	91.55% (0.858–0.951)	89.66% (0.836–0.936)	0.81	-

Notes: Combined models were constructed by binary logistic regression using the continuous values of the corresponding biomarkers.

**Table 5:** Sensitivity analysis of diagnostic performance according to histological group and FIGO stage.

Histological Group	FIGO Stage	Case (n)	LDH AUC (95% CI)	CA125 AUC (95% CI)	NLR AUC (95% CI)	LDH+CA125 AUC (95% CI)	LDH+NLR AUC (95% CI)	LDH+CA125+NLR AUC (95% CI)
HGSOC	Early I–II	28	0.586 (0.475–0.697)	0.848 (0.766–0.929)	0.637 (0.520–0.755)	0.799 (0.700–0.897)	0.615 (0.504–0.726)	0.809 (0.713–0.905)
HGSOC	Advanced III–IV	116	0.782 (0.725–0.839)	0.920 (0.880–0.959)	0.756 (0.699–0.813)	0.969 (0.950–0.987)	0.820 (0.769–0.872)	0.969 (0.950–0.987)
non-HGSOC	Early I–II	64	0.636 (0.556–0.717)	0.657 (0.567–0.747)	0.649 (0.567–0.730)	0.687 (0.608–0.765)	0.648 (0.568–0.727)	0.697 (0.619–0.775)
non-HGSOC	Advanced III–IV	30	0.729 (0.609–0.849)	0.916 (0.846–0.985)	0.739 (0.636–0.842)	0.927 (0.874–0.981)	0.746 (0.629–0.862)	0.936 (0.891–0.981)

Note: Sensitivity analyses were restricted to patients with complete LDH, CA125, and NLR data. All EOC subgroups were compared with the same benign ovarian lesion reference group (*n* = 238). Non-HGSOC included low-grade serous, endometrioid, clear cell, mucinous carcinomas, and ovarian carcinosarcoma. Combined models were based on continuous biomarker values entered into logistic regression models; triple-positive status was not used in this analysis.

**Table 6:** Model specifications, internal validation, and incremental diagnostic value of combined biomarker models for epithelial ovarian cancer.

**Panel A. Model Specifications and Internal Validation of the Combined Diagnostic Models for Epithelial Ovarian Cancer**									
**Cohort**	**Model**	**Intercept**	**βLDH**	**βCA125**	**βNLR**	**Probability** **threshold**	**Apparent** **AUC**	**Optimism**	**Bootstrap-corrected** **AUC**
Overall	LDH+CA125	−5.148	0.021	0.014	—	0.465	0.867	0.001	0.867
Overall	LDH+NLR	−4.394	0.020	—	0.173	0.569	0.759	0.002	0.757
Overall	LDH+CA125+NLR	−5.169	0.020	0.014	0.071	0.437	0.873	0.003	0.870
Early stage	LDH+CA125	−3.707	0.017	0.013	—	0.421	0.727	0.005	0.723
Early stage	LDH+NLR	−3.189	0.015	—	0.165	0.464	0.673	0.010	0.664
Early stage	LDH+CA125+NLR	−3.873	0.015	0.013	0.156	0.500	0.751	0.012	0.738
Advanced stage	LDH+CA125	−8.249	0.033	0.017	—	0.349	0.958	0.001	0.957
DAdvanced stage	LDH+NLR	−5.361	0.024	—	0.183	0.544	0.817	0.002	0.816
Advanced stage	LDH+CA125+NLR	−8.229	0.033	0.017	0.017	0.329	0.958	0.004	0.954
**Panel B. Incremental diagnostic value of NLR when added to the LDH+CA125 model**									
**Cohort**	**LDH+CA125** **AUC**	**LDH+CA125+NLR** **AUC**	**ΔAUC**	**DeLong P**	**LR χ^2^**	**LR-test P**	**Sensitivity, % (2-marker →3-marker)**	**Specificity, % (2-marker** **→ 3-marker)**	
Overall	0.867	0.873	0.005	0.092	3.539	0.060	73.39 → 75.97	89.92 → 88.66	
Early stage	0.727	0.750	0.023	0.157	5.522	0.019	73.91 → 61.96	65.59 → 83.87	
Advanced stage	0.958	0.958	−0.000	0.778	0.207	0.649	90.85 → 91.55	91.03 → 89.66	

Note: Combined diagnostic models were constructed by binary logistic regression using continuous biomarker values. Separate models were fitted for the overall, early-stage, and advanced-stage cohorts. The probability threshold for each model was determined by maximizing the Youden index in the development dataset. Internal validation was performed using 1000 bootstrap resamples, and optimism-corrected AUCs are reported.

**Table 7:** Univariable and multivariable logistic regression analyses of factors associated with platinum resistance in EOC.

Univariable Analysis				Multivariable Analysis			
Variable	OR	95% CI	p Value	Variable	Adjusted OR	95% CI	p Value
Age (>50 vs. ≤50 years)	1.210	0.493–2.968	0.677				
BMI (non-normal vs. normal)	1.071	0.502–2.286	0.860				
LDH (high vs. low)	3.023	1.248–7.326	0.014				
CA125 (high vs. low)	2.122	0.778–5.787	0.141				
NLR (high vs. low)	2.519	0.990–6.413	0.053				
Triple-positive status	4.455	2.012–9.861	<0.001	Triple-positive status	3.488	1.504–8.087	0.004
Primary debulking surgery (PDS vs. NACT–IDS)	2.651	0.603–11.667	0.197				
Tumor distribution (non-unilateral vs. unilateral)	1.210	0.556–2.633	0.631				
Tumor size (≥10 vs. <10 cm)	2.380	0.875–6.477	0.090				
Pelvic peritoneal dissemination	2.058	0.950–4.460	0.067				
Pleural effusion	1.970	0.390–9.947	0.412				
Ascites	3.703	1.695–8.090	0.001	Ascites	2.697	0.986–7.379	0.053
Lymph node dissection	0.863	0.374–1.989	0.729				
R0 resection	0.652	0.246–1.730	0.390				
Pathological grade (high vs. low/moderate)	1.980	0.809–4.847	0.135				
Histological subtype (serous vs. non-serous)	1.509	0.661–3.442	0.328				
Vascular tumor thrombus	1.732	0.791–3.797	0.170				
Lymph node metastasis	1.441	0.406–5.119	0.572				
Positive ascites cytology	2.596	1.204–5.599	0.015				
FIGO stage (III–IV vs. I–II)	3.021	1.189–7.678	0.020	FIGO stage (III–IV vs. I–II)	1.057	0.311–3.593	0.930

**Table 8:** Univariate and multivariate Cox regression analysis of factors associated with PFS in EOC.

Univariable Analysis				Multivariable Analysis			
Variable	HR	95% CI	p Value	Variable	Adjusted HR	95% CI	p Value
Age (>50 vs. ≤50 years)	0.695	0.408–1.184	0.181				
BMI (non-normal vs. normal)	1.166	0.712–1.910	0.541				
LDH (high vs. low)	1.564	0.948–2.581	0.080				
CA125 (high vs. low)	2.018	1.241–3.282	0.005				
NLR (high vs. low)	1.315	0.779–2.219	0.305				
Triple-positive status	4.453	2.659–7.457	<0.001	Triple-positive status	3.109	1.772–5.456	<0.001
Primary debulking surgery (PDS vs. NACT–IDS)	0.779	0.397–1.529	0.468				
Tumor distribution (non-unilateral vs. unilateral)	1.669	1.032–2.700	0.037	Tumor distribution (non-unilateral vs. unilateral)	1.027	0.621–1.700	0.917
Pelvic mass size ≥ 10 cm	1.460	0.903–2.362	0.123				
Pelvic peritoneal dissemination	1.419	0.878–2.293	0.153				
Hepatic metastasis	0.775	0.282–2.129	0.621				
Splenic metastasis	1.291	0.405–4.113	0.666				
Pleural effusion	1.127	0.275–4.617	0.868				
Ascites	2.863	1.769–4.634	<0.001	Ascites	2.208	1.181–4.126	0.013
Lymph node dissection	0.860	0.501–1.476	0.583				
R0 resection	0.729	0.381–1.392	0.338				
Pathological grade (high vs. low/moderate)	3.504	1.866–6.583	<0.001	Pathological grade (high vs. low/moderate)	2.235	1.075–4.646	0.031
Histological subtype (serous vs. non-serous)	1.780	1.033–3.069	0.038				
Vascular tumor thrombus	1.007	0.623–1.626	0.979				
Lymph node metastasis	1.108	0.624–1.969	0.726				
Positive ascites cytology	1.115	0.682–1.822	0.665				
FIGO stage (III–IV vs. I–II)	2.284	1.315–3.967	0.003	FIGO stage (III–IV vs. I–II)	0.728	0.341–1.554	0.412

**Table 9:** Univariate and multivariate Cox regression analysis of factors associated with OS in patients with advanced-stage EOC.

Univariable Analysis				Multivariable Analysis			
Variable	HR	95% CI	p Value	Variable	Adjusted HR	95% CI	p Value
Age (>50 vs. ≤50 years)	0.887	0.383–2.053	0.779				
BMI (non-normal vs. normal)	0.725	0.356–1.475	0.374				
LDH (high vs. low)	1.464	0.676–3.170	0.334				
CA125 (high vs. low)	1.758	0.831–3.717	0.140				
NLR (high vs. low)	1.980	0.814–4.815	0.132				
Triple-positive status	4.378	1.795–10.678	0.001	Triple-positive status	3.538	1.385–9.038	0.008
Primary debulking surgery (PDS vs. NACT–IDS)	0.584	0.250–1.360	0.212				
Tumor distribution (non-unilateral vs. unilateral)	1.568	0.782–3.142	0.205				
Pelvic mass size ≥10 cm	2.221	1.095–4.505	0.027	Pelvic mass size ≥ 10 cm	1.350	0.643–2.835	0.428
Pelvic peritoneal dissemination	1.862	0.803–4.315	0.147				
Hepatic metastasis	0.220	0.030–1.613	0.136				
Splenic metastasis	1.040	0.248–4.357	0.958				
Pleural effusion	1.842	0.437–7.764	0.405				
Ascites	2.328	1.075–5.040	0.032	Ascites	1.988	0.906–4.360	0.086
Lymph node dissection	1.065	0.491–2.311	0.873				
R0 resection	0.833	0.359–1.930	0.670				
Pathological grade (high vs. low/moderate)	1.233	0.505–3.009	0.646				
Histological subtype (serous vs. non-serous)	0.716	0.293–1.747	0.463				
Vascular tumor thrombus	0.913	0.410–2.035	0.824				
Lymph node metastasis	0.496	0.214–1.151	0.103				
Positive ascites cytology	1.078	0.532–2.184	0.835				

## Data Availability

The data presented in this study are available from the corresponding author upon reasonable request. The data are not publicly available because they contain clinical information that is subject to patient privacy and ethical restrictions.

## Abbreviations

The following abbreviations are used in this manuscript:

OC	Ovarian cancer
EOC	Epithelial ovarian cancer
LDH	Lactate dehydrogenase
CA125	Carbohydrate antigen 125
NLR	Neutrophil-to-lymphocyte ratio
BMI	Body mass index
HRT	Hormone replacement therapy
CEA	Carcinoembryonic antigen
CA19-9	Carbohydrate antigen 19-9
AFP	Alpha-fetoprotein
HE4	Human epididymis protein 4
HGSOC	High-grade serous ovarian carcinoma
ROC	Receiver operating characteristic
AUC	Area under the receiver operating characteristic curve
CI	Confidence interval
OR	Odds ratio
HR	Hazard ratio
PFS	Progression-free survival
OS	Overall survival
FIGO	International Federation of Gynecology and Obstetrics
RECIST	Response Evaluation Criteria in Solid Tumors
PDS	Primary debulking surgery
NACT–IDS	Neoadjuvant chemotherapy followed by interval debulking surgery
R0	No macroscopic residual disease
RMI	Risk of Malignancy Index
ROMA	Risk of Ovarian Malignancy Algorithm
BRCA1/2	Breast cancer susceptibility genes 1 and 2
HRD	Homologous recombination deficiency
